# Systemic therapies for salivary gland carcinomas: an overview of published clinical trials

**DOI:** 10.4317/medoral.26264

**Published:** 2023-12-27

**Authors:** Luan César Silva, Maria Eduarda Pérez-de-Oliveira, Caique Mariano Pedroso, Amanda Almeida Leite, Alan Roger Santos-Silva, Marcio Ajudarte Lopes, Gilberto de Castro Junior, Manoela Domingues Martins, Vivian Petersen Wagner, Luiz Paulo Kowalski, Cristiane Helena Squarize, Rogerio Moraes Castilho, Pablo Agustin Vargas

**Affiliations:** 1 Department of Oral Diagnosis, Piracicaba Dental School, University of Campinas, Piracicaba, São Paulo, Brazil; 2 Laboratory of Epithelial Biology, Department of Periodontics and Oral Medicine, University of Michigan School of Dentistry, Ann Arbor, Michigan, USA; 3 Serviço de Oncologia Clínica, Instituto do Câncer do Estado de São Paulo, Hospital das Clinicas HCFMUSP, Faculdade de Medicina, Universidade de São Paulo, São Paulo, SP, Brazil; 4 Department of Oral Pathology, School of Dentistry, Federal University of Rio Grande do Sul, Porto Alegre, Rio Grande do Sul, Brazil; 5 Department of Oral Pathology, School of Clinical Dentistry, The University of Sheffield-UK; 6 Department of Head and Neck Surgery and Otorhinolaryngology, A C Camargo Cancer Center, São Paulo, SP, Brazil

## Abstract

**Background:**

There is no consensus about effective systemic therapy for salivary gland carcinomas (sgcs). Our aim was summarized the clinical trials assessing the systemic therapies (ST) on sgcs.

**Material and Methods:**

Electronic searches were carried out through MEDLINE/pubmed, EMBASE, Scopus, Web of Science, and the Cochrane Library databases, and gray literature.

**Results:**

Seventeen different drugs were evaluated, and the most frequent histological subtype was adenoid cystic carcinoma (*n*=195, 45.5%). STable disease, observed in 11 ST, achieved the highest rate in adenoid cystic carcinoma treated with sunitinib. The highest complete (11.1%) and partial response (30.5%) rates were seen in androgen receptor-positive tumors treated with leuprorelin acetate.

**Conclusions:**

Despite all the advances in this field, there is yet no effective evidence-based regimen of ST, with all the clinical trials identified showing low rates of complete and partial responses. Further, translational studies are urgently required to characterize molecular targets and effective ST.

** Key words:**Clinical trials, salivary gland cancer, systemic therapies.

## Introduction

Salivary gland carcinomas (SGCs) are a rare type of cancer, comprising approximately 3-6% of all head and neck cancers (1,2). The management of SGCs is based on the tumor stage and the histopathological subtype, with radical surgical resection remaining the standard treatment for early-stage disease, and radiotherapy may be recommended as adjuvant therapy (3). Currently, the role of systemic therapies in SGCs is palliative, and generally indicated in the advanced/metastatic setting, with low (< 10%) response rates for cytotoxic agents. In addition, a few patients may derive clinical benefits from hormonal therapies, anti-angiogenics, and immunotherapy (4-7).

Given the SGCs complexity, robust preclinical models are being developed to advance basic and translational research within the field; however, the mixed results presented by these studies have delayed new clinical studies (8). At present, there is no consensus about the best regimen of systemic therapy for SGCs. Keeping this in mind, our review aimed to integrate and summarize existing clinical trial studies that assessed the use of systemic therapies for treating SGCs.

## Material and Methods

This review was based on the following review question: “What are the currently available systemic therapies for treating salivary gland carcinomas and their response rates?”. Individualized search strategies were developed for each of the following electronic databases with all the searches being undertaken on May 5th, 2022: PubMed/MEDLINE, Scopus, Embase, Web of Science, and the Cochrane Library. Furthermore, a gray literature search was performed including Google Scholar, Open Grey, and ProQuest (Supplement 1). The study design only assessed and summarize randomized/non-randomized clinical trials published in the English language, and studies that 1) not analyzed systemic therapies for SGC; 2) included other malignant tumor beyond that SGCs; 3) included reviews, case reports, case series, observational studies, protocols, short communications, personal opinions, letters, conference abstracts, and laboratory research; and 4) published in a language other than English or 5) the full text was not available, were excluded.

After database screening, we removed any duplicate articles using a reference management software (EndNote X7, Thomson Reuters, Philadelphia, PA, USA), and the selection process was performed in two phases by two independent authors (LCS and MEPO). In phase one, all articles underwent a preliminary screening of titles and abstracts using Rayyan® (Rayyan, Qatar Computing Research Institute, Doha, Qatar) to determine whether they met the inclusion criteria or not. In phase two, the full texts of all selected articles in phase one were read applying the same eligibility criteria and recording the exclusion reasons. Disagreements between the two initial evaluators were resolved by consensus. If a consensus could not be reached, a third reviewer (CMP) was involved to make the final decision.

The following data were extracted from each included article (when available): publication information (reference, country, year of publication), sample information (sample size; patient sex, age, and race; site of primary disease; histologic subtypes; clinical stage; performance status; prior treatments; recurrence; presence of locoregional and/or distant metastases and metastatic site involvement), systemic therapy information (drug, dose/scheme, administration route, treatment response evaluation, tumor size regression, complete and partial responses, sTable disease, duration of responses, progression of disease, time to disease progression) survival information (survival times and rates, mean follow-up period, and patients’ vital status), and main conclusions.

A qualitative analysis was performed by grouping similar data from all included studies to describe the frequency for each variable and their categories.

## Results

A total of 1,274 articles were identified in the five main databases in the three gray texts. After removing 905 duplicated articles, 522 remained for reading of their titles and abstracts (phase one). In phase two, 44 articles were selected, being 29 articles excluded by a careful reading of the full texts considering the eligibility criteria, and the remaining 15 articles were included. The reasons for the exclusion of the articles read in full are presented in Supplement 2.

**Figure 1:**
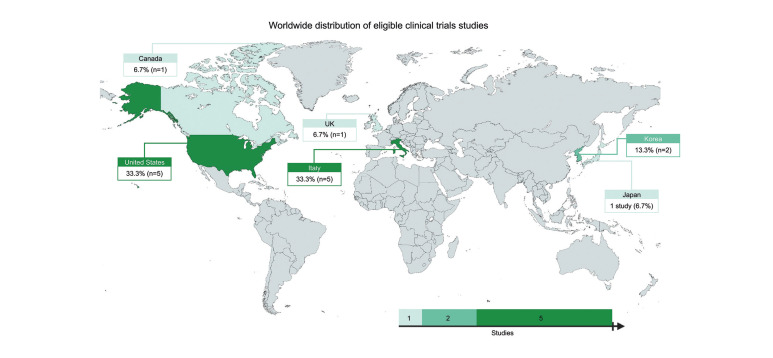
World map showing the distribution of eligible clinical trials studies (frequency in %).

The 15 articles included in this review were published between 1991 and 2021, with most (n = 13; 86.7%) being published after 2001. These studies were performed in six different countries: five studies from Italy (9-13), five from the USA(14-18), two from Korea (19,20), one from Japan (21), one from Canada (22), and one from the United Kingdom (23) (Fig. 1).

## Discussion

- Clinicopathological profile of salivary gland carcinomas

The clinicopathological features of all studied SGCs are summarized in Table 1. The sample size of included studies ranged from 13 to 60 patients, comprising a total of 422 patients diagnosed with SGCs, being 278 males and 144 females (male:female ratio of 1.93:1). The age of patients ranged from 19 to 90 years, although Jones *et al*., 1993 reported the mean age without maximal and minimal ages. Four (26.7%) articles (14,15,17,18)(n = 108, 25.6%) described the patients’ race, with most patients being classified as white (n = 93, 86.1%) and Asian (*n*=7, 6.5%).

**Table 1:**
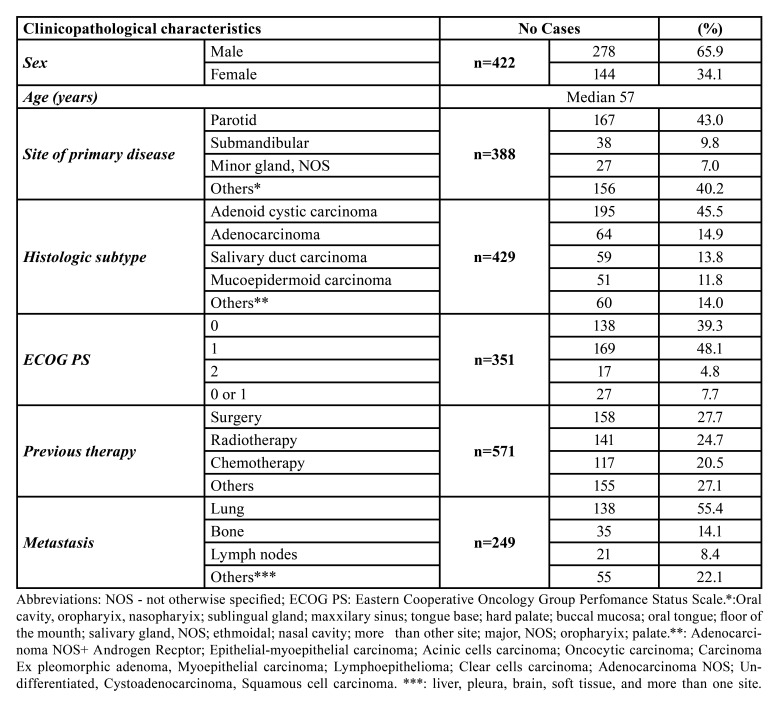
Clinicopathological characteristics of 422 cases of salivary gland carcinomas.

The site of primary disease was described in all studies, except in Kim *et al*. (2017) and Chau *et al*. (2012) which only described the site as the head and neck area (*n*=20, 4.7%) and the major or minor salivary glands (*n*=14, 3.3%) without further specification, respectively. The most affected site of primary disease was parotid gland (*n*=167, 43.0%), followed by submandibular gland (n = 38, 9.8%). The most reported malignant tumor was adenoid cystic carcinoma (ACC) (n = 195; 45.5%) followed by adenocarcinoma (the authors did not indicate the specific diagnosis) (n = 64; 14.9%), and salivary duct carcinoma (n = 59; 13.8%). Here, it is important to highlight the fact that the sum of all histological subtypes exceeds the overall sample size (*n*=429) because Airoldi, *et al*. (2017) described seven additional cases and the authors did not explain in the manuscript why the sum of all histological subtypes was more than their sample size.

The patients’ performance status was described in most studies (*n*=351, 83.2%) using the Eastern Cooperative Oncology Group (ECOG) scale, with 138 (39.3%) cases being classified as 0; 169 cases (48.1%) classified as 1; and 17 cases (4.8%) classified as 2. Two publications (16,17) reported the ECOG performance in a group of scores 0 and 1 (n = 27 patients; 7.7%). Three articles did not describe performance status (11,12,23). Further, it is important to highlight witch 571 tumors received prior treatments before the respective trial (surgery: *n*=158, 27.7%; radiotherapy: *n*=141, 24.7%; and chemotherapy: *n*=117, 20.5%). Twenty-eight (6.6%) patients did not receive any previous treatment (11,15,19,21). Only one article (*n*=16, 3.8) did not report previous therapies (23).

Metastatic disease was present in 249 out of 422 patients (9-13,15,19-21). The main metastatic sites were the lung (*n*=138, 55.4%), bone (*n*=35, 14.1%), and lymph nodes (*n*=21). Only Locati, *et al*., 2009 did not report all metastatic sites. Seven articles (*n*=163, 38.6%) did not indicate the site of metastasis (15-18,21-23). Six articles (*n*=106, 25.1%) did not report any metastasis (14,16-18,22,23).

- Systemic therapeutic approaches aiming salivary gland carcinomas

Emerging studies have investigated molecular targets to avoid the tumor resistance found in conventional therapies (platinum-based chemotherapy) (24). High levels of c-Kit expression were seen in ACC, however, when used as target therapy, no objective response was achieved (8). Epigenetic events have been shown to “in vitro” play a role in MEC behavior (25-28), while a phase II trial demonstrated that vorinostat, a histone deacetylase inhibitor, had accepTable efficacy in ACC patients (29). However, there has been no phase II study that exclusively assessed MEC (30). Furthermore, some advance has been made with FDA approved drugs, such as NRTK inhibitors (larotrectinib) for NTRK-fusion positive tumors (31), which demonstrated robust and durable activity in SGC, including secretory carcinoma (32). Keep this in mint, we assessed and integrated the available evidence about systemic therapies published in the English literature to treat SGCs.

The systemic therapeutic approaches and their respective treatment outcomes are summarized in Table 2. Eighteen systemic therapies schemes were evaluated comprising seventeen different drugs. Three different clinical trials evaluated the cisplatin + vinorelbine combination (9,10,19) and cisplatin alone was evaluated by two other trials (11,23). The most common administration routes were intravenous (n = 10 articles) and oral (n = 6 articles). The number of cycles ranged from 1 to 54 cycles; however, one study did not report this information (21) and two articles only described the median treatment duration in months (13,17).

SGCs are among the most complex head and neck tumors due to the heterogeneity of their population cells (33), and that makes them challenging to treat. In general, surgery is the main treatment option with curative intent of early-stage SGCs (7), as observed in our findings, in which 27.7% of the cases were previously treated by surgical resections. In addition, a review by Sahara *et al*., 2021 showed that radiation therapy also has a positive effect on local disease control, but its effect on prolonging overall survival is unclear. On the other hand, several chemical agents have been investigated in SGCs, such as target therapy for vascular endothelial growth factor receptor (VEGF) and epidermal growth factor receptor (EGFR) signaling in ACC, and histone deacetylase and the mammalian target of rapamycin (mTOR) in MEC (30). However, there were no randomized controlled trials with adequate power comparing the use of different systemic therapy protocols in SGCs.

The treatment response was evaluated in almost all studies (10,12-14,17-22) (according to the Response Evaluation Criteria in Solid Tumors (RECIST) guidelines. The most prevalent outcome observed was sTable disease (SD) in studies evaluating the following agents: (tipifarnib, pembrolizumab + vorinostat, leuprorelin acetate, vinorelbine + cisplatin, vinorelbine alone, nintedanib, sunitinib, bortezomib + doxorubicin, bortezomib, cetuximab, and epirubicin + 5-fluoracil), with the highest SD rate observed in patients diagnosed with ACC and treated with sunitinib while the lowest was seen in SGCs treated with epirubicin + 5-fluoracil. In addition, complete response was seen just in some articles, and with low rates of response. This happened after the treatment with the following agents: tipifarnib (7.7%), leuprorelin acetate + bicalutamide (11.1%), vinorelbine + cisplatin (6%), and cisplatin alone (7.3%).

Usually, systemic therapies were administered in combinations of agents with distinct mechanisms of action.

**Table 2:**
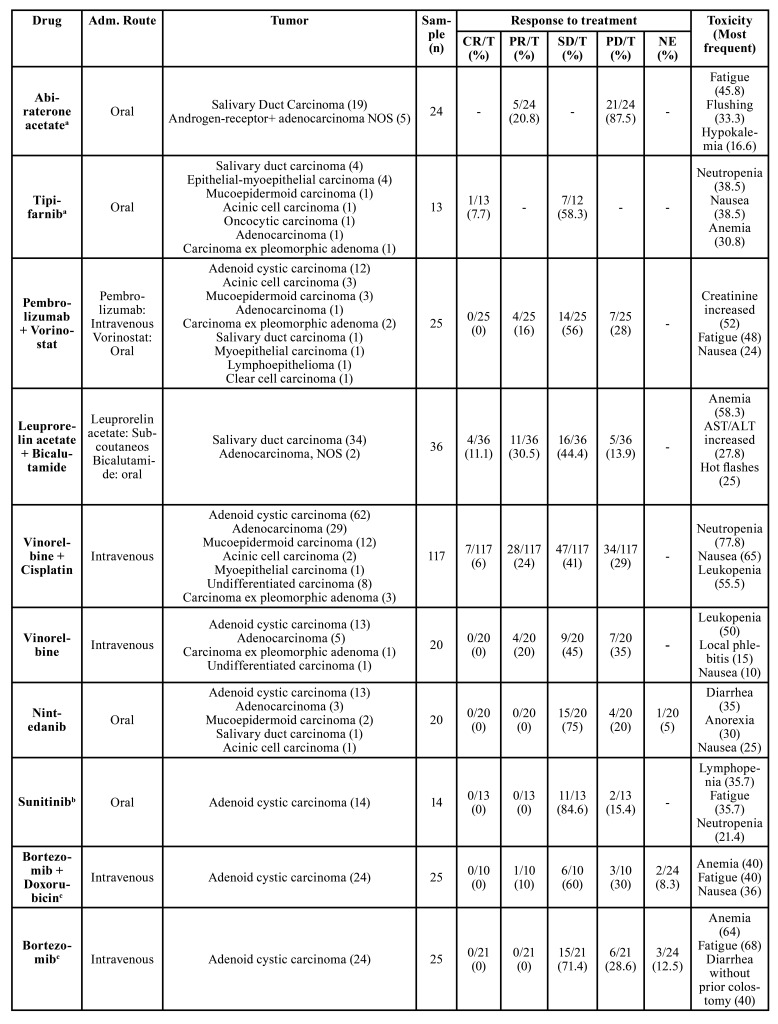
The 15 studies that evaluated the systemic therapies for advanced salivary gland carcinomas and their administration route, responses, and most toxicities.

**Table 2 cont.**
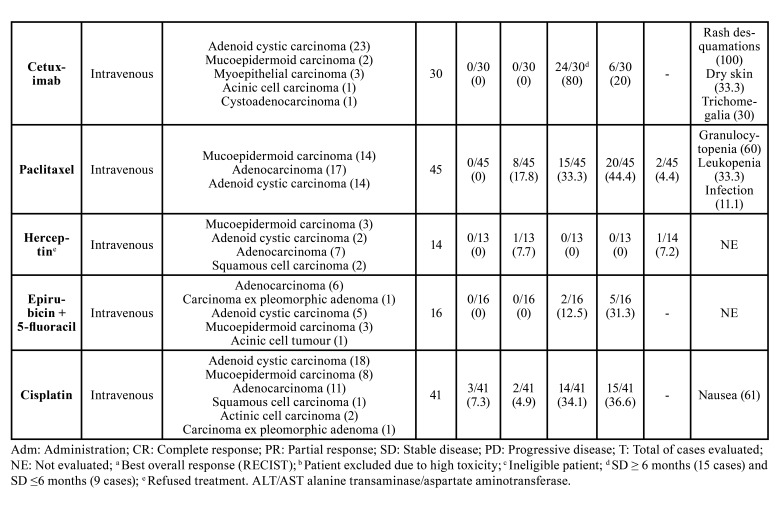
The 15 studies that evaluated the systemic therapies for advanced salivary gland carcinomas and their administration route, responses, and most toxicities.

Here, we found that despite pembrolizumab + vorinostat, vinorelbine + cisplatin, vinorelbine alone, bortezomib + doxorubicin, epirubicin + 5-fluoracil presenting partial response (PR), the most prevalent positive responses observed were SD, while abiraterone acetate, paclitaxel, and cisplatin for progressive disease. Also, the lack of effective systemic treatment options for advanced SGCs, could explain the fact of it is not uncommon to find metastasis as the most common type of relapse in SGC. In this review, approximately 60% presented as metastatic cases, and the most frequent was metastasis in the lung (55.4%). However, it is hard to predict the best treatment to SGCs, once the main studies included in this review, analyzed a small number of patients, included different histopathological subtypes into a one and only group, and did not specify the objective response to each tumor. Finally, it is important to report that some articles included other malignant tumors beyond that SGCs, like clinical trials evaluating the imatinib mesylate for adenoid cystic carcinoma (34,35). And for this reason, these articles were not included in the present review.

- Toxicity of systemic treatment of salivary gland carcinomas

Side effects and toxicities were described in almost all studies, except in those by Jones *et al*. (1993) and Haddad *et al*. (2003). The most common side effects seen were nausea (*n*=173), neutropenia (*n*=172), leukopenia (*n*=67), and fatigue (*n*=61) (Table 2).

It is very important to consider toxicity in patients with SGCs. Fatigue, nausea, neutropenia, and leukopenia were the main toxicities found in this current review. On the other hand, we know that other therapies could imply severe side effects, when patients are frequently submitted to large surgical resections (mainly in large tumors), or radiotherapy, presenting anatomic and functional sequelae, suggesting that this approach should not be recommended as a palliative treatment. Therefore, its necessary to consider when the only remaining therapeutic modality for palliative care is based on drugs.

- Survival and outcomes of systemic treatment of salivary gland carcinomas

Overall survival (OS) was reported in 11 articles (9-11,13-15,17-19,21,22) and progression free-survival (PFS) was reported in eight studies (10,13,14,17-19,21,22). The highest median OS and PFS were observed in the study by Fushimi *et al*., 2018, that evaluated the use of leuprorelin acetate + bicalutamide combination in androgen receptor-positive patients. The lowest median OS and PFS were seen in the clinical trial conducted by Airoldi *et al*., 2017, in which patients treated with a cisplatin + vinorelbine combination as a second line treatment have a median survival of four months. The median follow-up time of patients ranged from 243 days (23) to 60.7 months (15). Patients’ vital status at last contact was described in six studies (n = 112, 26.5%), in which 70 (62.5%) patients were dead and 42 (37.5%) were alive with persistent disease. However, Gilbert *et al*., 2006 only reported one treatment-related death and did not detail the status of the other patients.

## Conclusions

Some limitations of this review should be highlighted. First, although a few clinical trials have been conducted on this topic, they usually have small and heterogeneous samples, divergent results, and no control group. Second, most of the responses to the treatments are presented together, as a single group of tumors, and do not describe the specific response of each one, which severely impacts our ability to provide a high-quality overall synthesis of the evidence. Retrieving the original data from all these studies in an international collaborative effort has the potential to produce a more informative analysis. Finally, the rarity of these tumors could be compromised the study design and randomized of included studies.

In conclusion, based on the currently available evidence, it is not possible to make an evidence-based judgement on the effectiveness of systemic therapy. Translational studies are, therefore, urgently needed to better characterize new molecular drivers in these tumors, either histopathologic-specific or agnostic, to develop more effective molecular-targeted systemic treatments which would allow well-designed clinical trials with a higher probability of success to be conducted.
